# Effect of coronary artery bypass grafting on blood pressure response to head-up tilting

**DOI:** 10.1186/s12576-020-00746-1

**Published:** 2020-03-30

**Authors:** Shinnosuke Hori, Yoshi-ichiro Kamijo, Mitsuru Yuzaki, Tetsuya Kawabe, Kohei Minami, Yasunori Umemoto, Mao Yokoyama, Hiroyasu Uenishi, Yoshiharu Nishimura, Ken Kouda, Yukio Mikami, Fumihiro Tajima

**Affiliations:** 1 Department of Rehabilitation Medicine, Wakayama Medical University, School of Medicine, 811-1 Kimiidera, Wakayama, 641-8509 Japan; 2 Department of Thoracic and Cardiovascular Surgery, Wakayama Medical University, School of Medicine, 811-1 Kimiidera, Wakayama, 641-8509 Japan; 3 Department of Center for Educational Research and Development, Wakayama Medical University, School of Medicine, 811-1 Kimiidera, Wakayama, 641-8509 Japan; 4 Department of Cardiovascular Medicine, Wakayama Medical University, School of Medicine, 811-1 Kimiidera, Wakayama, 641-8509 Japan

**Keywords:** Body fluid, Myocardial infarction, Angina pectoris, Rehabilitation

## Abstract

Blood pressure response to head-up tilt (HUT) in 7 healthy subjects and 9 patients before and after coronary artery bypass grafting (CABG) was measured during supine and 15-min 60° HUT. Stroke volume (SV) and ejection fraction (EF) were assessed by echocardiography. Baseline mean arterial pressure (MAP) and heart rate (HR) in patients before CABG were similar to healthy subjects. MAP in patients decreased by 6 (4–9) mmHg [median (1st–3rd quartiles)] during 7–12 mmHg of HUT with decreased cardiac output (CO = SV × HR) while HR remained unchanged. MAP in healthy subjects remained unchanged during HUT with increased HR. Body weight decreased by 3.5 (2.5–3.7) kg and MAP decreased by 6 (2–13) mmHg during the last 3-min HUT while HR increased after CABG. Decreases in SV and CO during HUT disappeared after CABG. Blood pressure decreased during HUT in patients before and after CABG regardless of HR response.

## Introduction

Coronary artery bypass grafting (CABG) for patients with coronary artery disease (CAD) has long-term benefits compared to other treatments, such as taking medications and percutaneous coronary intervention with drug-eluting stent [1]. About 20,000 CABG were performed at 1573 facilities in 2016 in Japan [2]. Rehabilitation therapy accelerates participation in society in patients with CAD mediated by improvements of their physical capacity [3]. Early rehabilitation therapy after cardiac surgery prevented patients from postoperative complications and improved their functional capacity and reduced length of hospital stay [4].

Rehabilitation program includes walking and/or ergometer exercise [5]. About 30% of patients after myocardial infarction develop syncope during the head-up tilt (HUT); during which systolic blood pressure drops to < 70 mmHg with and without decrease in heart rate (HR) to < 40 beats/min, a rate significantly higher than age-matched healthy male volunteers (10%) [6]. Another cohort study showed that the incidence of orthostatic hypotension after cardiac surgery was ~ 30% during early mobilization [7], which was higher than individuals with normal cardiovascular function.

A change in posture from supine to standing generally reduces venous return to the heart with associated unloading of baroreceptors. This sets a reflex increase in HR and total peripheral vascular resistance (TPR) through the autonomic nervous system to maintain arterial blood pressure (BP) [8]. Several studies have shown increased sympathetic and decreased parasympathetic tone in patients with acute myocardial infarction at 30 or 60 days after CABG, compared with age-matched healthy subjects [9, 10]. However, the sensitivity of HR response for a given change in BP was attenuated in CAD patients, such as myocardial infarction [11] and angina pectoris [12] and is considered a significant prognostic factor in such patients [13]. In addition, patients who undergo cardiac surgery are often treated with β-blocker, αβ-blocker, calcium antagonist, angiotensin II receptor blocker and nitrates to prevent the development of postoperative atrial fibrillation [14, 15] and recurrent myocardial infarction [16, 17]. Diuretics keeps from causing fluid overload, which induces heart failure [17] and acute lung and kidney injuries [18], and making cardiac surgery patients to stay hospital for a longer length [19]. Extravasation of intravascular fluid through the vascular endothelium is common on the 1st day after cardiac surgery due to an enhancement of endothelium permeability [20]. When vascular permeability is restored 2 to 3 days after surgery, free water moves back to the blood vessels; it called as “refilling”. Therefore, adequate fluid resuscitation is needed during the first 2- or 3-day post operation, then diuretics generally are prescribed intermittently or continuously after surgery. Lower plasma volume in postoperative phase is concerned. Such patients could be at risk of orthostatic hypotension during rehabilitation therapy.

One previous study examined effects of HUT in patients who had undergone CABG 3 months after and showed no decrease in systolic- and diastolic blood pressure during HUT [21]; however, there is no study that examined them within a shorter postoperative period. The purpose of the present study was to determine differences in orthostatic BP response and cardiac function between patients scheduled for CABG surgery and healthy subjects, and in the patients before and after CABG followed by rehabilitation therapy. Our hypotheses were that (1) BP would decrease by HUT in the patients before CABG while no significant decrease was observed in healthy subjects and (2) the decrease in BP would still be recognized after CABG even cardiac function improved more than before surgery.

## Methods

### Subjects

Subjects were 7 male and 2 female patients with CAD who were admitted to an acute care hospital between January 2017 and June 2018 for an isolated CABG and had no histories of chronic obstructive pulmonary disease, stroke, or severe cardiac arrhythmia, and 7 age- and physical characteristics-matched healthy subjects involved one female. All healthy subjects were free from diabetes mellitus, neurological, cardiovascular, and respiratory disorders. The present procedures are in accordance with the Helsinki Declaration as revised in 2013 and were approved by the Ethics Committee of our institution. Informed consent was obtained from all individual participants included in the study before participation. Additional informed consent was obtained from all individual participants for whom identifying information is included in this article.

### Rehabilitation program

Patients were transferred after CABG to the intensive care unit and received ventilatory support in addition to treatment for hemodynamic stabilization and electrolyte balance. Rehabilitation therapy commenced on the first day after CABG depending on their condition. On postoperative day (POD) 1, patients were mobilized out of bed to perform resistance training for lower lib muscles. On POD 2–3, they performed resistance exercise, exercises of daily living, standing position, and gait training. On POD 4–5, the distance and frequency of walking exercise were increased taking into consideration of their condition every day except for Sunday. They exercised 60 min per session with treadmills and/or cycle ergometers at 50 to 70% of peak HR and/or perceived exertion of between “light” and “somewhat hard” (12 to 14 of a Borg Scale rating). After POD 6, they performed the same exercise as POD 4–5 with treadmill and/or cycle ergometer for 60 min per session and work load was adjusted every day, so that the HR at the first 5 min of exercise was equivalent to their target HR and/or 12 to 14 of a Borg Scale rating. They additionally performed a stair climbing within the sessions if they were not exhausted. All patients achieved whole program during admission period.

### Protocol

This was a cross-sectional (patients before surgery vs. healthy subjects) and a prospective observational study (before vs. after surgery). Experiments were performed 1–3 days before CABG and repeated 15 ± 7 days [mean ± SD] (8–29; min–max) after CABG just before discharged. All subjects reported to our rehabilitation room (25 °C) at 17:00 on the study day. Body weight was measured while subjects were dressed in cotton underwear. They relaxed in supine position on a tilt table (K1430MN, MINATO, Osaka) for 20 min. Surface electrodes were attached for recording ECG, a cuff was wrapped around the left upper arm for measurement of BP, and the left shoulder joint was fastened at abduction at 90° angle on a shelve fixed to the table, to keep the arm at heart level. The abdomen and the thigh were held in place on the table using straps. The feet were supported with board to prevent sliding down during HUT. The baseline measurements were recorded after another 10-min supine rest. This was followed by tilting the table to 60˚ within 40 s and maintained for 15 min, then returned to the supine position. None of the subjects experienced syncope or pre-syncope defined as dizziness and/or lightheadedness but not followed by loss of consciousness [22] due to the maneuvers.

### Measurements

#### Cardiovascular responses

Systolic (SBP) and diastolic blood pressures (DBP) were measured at 1-min interval throughout the study by the auscultatory method using a sphygmomanometer (STBP780, Colin, Tokyo). Mean arterial pressure (MAP) was calculated as (SBP − DBP)/3 + DBP. HR was calculated from mean *R*–*R* interval of ECG every minute. The signals of baseline were averaged for 10-min supine rest and those during HUT were averaged every 3 min (HUT3, HUT6, ..., HUT15; 5 levels of time point) for smoothing each signal.

#### Respiratory response

We controlled respiration rate at 0.5 Hz using a metronome in all subjects, while monitoring a respiratory movement with a piezoelectric belt transducer (MLT1132; ADInstruments, Colorado Springs, CO, US). Some patients hold their breath for about 5 s, when it is difficult to extract images.

#### Echocardiography

Transthoracic echo was performed using commercially available equipment (Vivid *i*; General Electric Health Care Japan, Tokyo) at 2.5-MHz cardiac sector ultrasound transducer probe. Experienced physicians, who were blinded to the clinical data, analyzed the echo images. Unfortunately, the echo data of one female patient recorded during HUT were not available. Therefore, we analyzed the echo data of 8 patients and 7 healthy subjects. Stroke volume (SV) and left ventricle ejection fraction (EF) were calculated by the method established previously [23, 24], at baseline, 1st and 15th min during HUT (HUT1 and HUT15, respectively), after assessing left ventricle end-diastolic (LVEDV) and end-systolic volumes (LVESV) by echocardiography. Cardiac output (CO) was calculated as SV multiplied by HR. TPR was expressed as MAP divided by CO using arbitrary units (mmHg/L/min).

#### Blood constituents

Serum albumin ([Alb]_s_, g/dL) and sodium ([Na^+^]_s_, mEq/L) concentrations and hematocrit (Ht, %) measured before and after surgery were obtained from the medical records as close to the study as possible. Albumin and sodium concentrations indicated colloid and osmotic pressures, respectively, and these were also indexes as dilution in blood.

### Statistical analysis

All values are expressed as median (1st–3rd quartiles) unless stated otherwise. All data were statistically analyzed using Shapiro–Wilk test. As a result, some data were not normally distributed. Therefore, non-parametric tests were conducted in the present study. Comparisons between healthy subjects and patients before surgery or after surgery at each time point: 3 levels for cardiac echo data (Baseline, HUT1 and HUT15); 6 levels for HR, MAP, SBP and DBP (Baseline and 5 levels every 3 min during HUT), were determined by the Mann–Whitney *U* test. Comparisons within subjects such as differences between before and after surgery in the patients and changes from “Baseline” to the time points during HUT: 3 levels for echo data and 6 levels for HR and BP, were assessed by Wilcoxon test. The null hypothesis was rejected when *P* < 0.05. SPSS version 24.0 (IBM SPSS Statistics, Chicago, IN) was employed in the analyses.

## Results

### Subjects’ characteristics

Age and physical characteristics of the patients (*n* = 9) and healthy subjects (*n* = 7) were 71 (68–78) and 65 (55–74) years (*P* = 0.13), 163 (160–172) and 164 (161–166) cm height (*P* = 0.36), 65 (60–72) and 60 (58–68) kg body weight (*P* = 0.34), and 25 (22–27) and 22 (22–25) body mass index (*P* = 0.35), respectively. After CABG, body weight decreased significantly in the patients by 3.5 (2.5–3.7) kg, compared with before surgery (*P* = 0.001). Baselines of SBP were 138 (133–149) mmHg and 123 (116–140) mmHg, DBP were 76 (66–80) mmHg and 76 (69–79) mmHg, and HR were 66 (59–71) beats/min and 74 (63–75) beats/min in the patients and healthy subjects, respectively, with no significant differences (all, *P* > 0.211). Baselines of MAP and SBP after CABG were 89 (88–90) mmHg and 121 (116–131) mmHg, significantly lower than before (all, *P* > 0.05), while DBP and HR were 70 (64–75) mmHg and 75 (61–84) beats/min with no significant differences from the values before CABG (all, *P* > 0.061). Table 1 lists the clinical diagnosis, complications, and severity classification for the patients. Table 2 shows numbers of patients in each medication before and after CABG. The duration of hospitalization was 17 ± 7 days [mean ± SD], which was follow-up period.

**Table 1:** Diagnosis, operative procedure, complications, and severity classification before surgery in 9 patients

ID	Diagnosis	Operative procedure	Grafts	Complications	NYHA
1	UAP	OPCAB	LITA-LA	HT	I
			RA-HL	HL	
2	UAP	OPCAB	LITA-LAD	HT	II
			GSV-OM-PL	HL	
			GSV-4PD		
3	OMI	OPCAB	RITA-LAD	HT	II
			LITA-OM	HL	
			GSV-4PD-4AV		
4	OMI	OPCAB	LITA-LAD	HT	I
			GSV-DG-OM	HL	
			GSV-PD-AV	DM	
5	OMI	OPCAB	LITA-LAD	HT	II
			RITA-DG	HL	
			GSV-OM	DM	
			GSV-AV		
			GSV-PD-AV		
6	OMI	CABG on-pump	LITA-LAD	HT	II
			RITA-DG	HL	
			GSV-HL-PL	DM	
			GSV-PD-AV		
7	UAP	OPCAB	LITA-LAD	HT	II
			GSV-OM-PL	HL	
			GSV-4PD-4AV	DM	
8	OMI	OPCAB	LITA-OM	HT	I
			RITA-LAD	HL	
			GSV-DG		
			RA-4PD		
9	OMI	OPCAB	RITA-LAD	HT	I
			GSV-PL-OM	HL	
			LITA-OM2	DM	

*UAP* unstable angina pectoris, *OMI* old myocardial infarction, *OPCAB* off-pump coronary artery bypass grafting, *HT* hypertension, *HL* hyperlipidemia, *DM* diabetes mellitus, *NYHA* New York Heart Association, *LAD* left anterior descending, *LITA* left internal thoracic artery, *RITA* right internal thoracic artery, *GSV* greater saphenous vein, *RA* radial artery, *HL* high lateral branch, *OM* obtuse marginal, *PL* posterolateral branch, *PD* posterior descending coronary artery, *AV* coronary arteriovenous, *DG* diagonal branch

**Table 2:** Numbers of patients in each medication

	β-B	αβ-B	Ca^2+^ ant	ARB	Nit	Cate	Diu	DA
Before	2	3	6	6	2	–	–	–
After	6	6	7	4	9	7	9	9

Before and After, before and after surgery; β-B, β-blocker; αβ-B, αβ-blocker; Ca^2+^ ant., calcium antagonist; ARB, angiotensin II receptor blocker; Nit., nitrates; Cate., Catecholamine; Diu., diuretics; DA, dopamine

### Differences between healthy subjects and patients

**Fig. 1:**
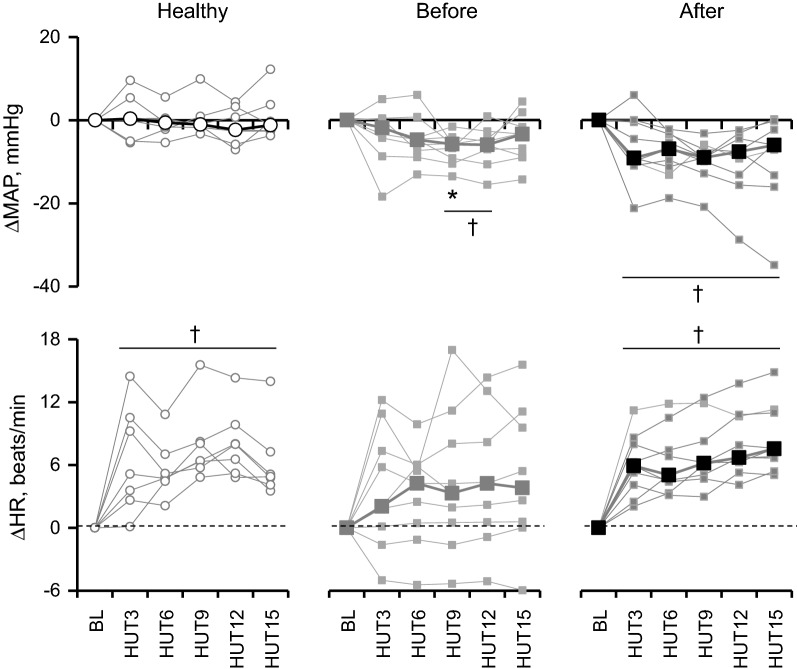
Changes (triangle) in mean arterial blood pressure (MAP) and heart rate (HR) from baseline (BL) in healthy subjects, patients, a***nd after surgery. Median and individual data for 7 healthy subjects (Healthy) and 9 patients before (Before) and after surgery (After) were shown. BL, supine before head-up tilt; HUT3, 6, 9, 12, and 15, averaged values from the onset of head-up tilt to 3 min, from 3 to 6 min, from 6 to 9 min, from 9 to 12 min and from 12 to 15 min, respectively. *, vs. Healthy; †, vs. BL at the level of *P* < 0.05

As shown in Tables 3 and 4 and Fig. 1, in healthy subjects, MAP remained unchanged during 60° HUT, but HR significantly increase during HUT compared from baseline (*P* < 0.05) and the increases were 5 (4–6) beats/min at HUT15 (*P* = 0.018; Fig. 1). SV, CO and TPR remained unchanged in this group (*n* = 7; *P* > 0.237). For patients (*n* = 9), MAP in the supine position before CABG was not significantly different from healthy subjects. However, it decreased at HUT9 and HUT12 compared from baseline (Table 3) and the decreases were 6 (6–9) (*P* = 0.008) and 6 (4–7) (*P* = 0.011), respectively, as shown in Fig. 1 [even median values were similar at HUT 12 (Table 3), the decrease was observed in all CABG subjects except one subjects (Fig. 1)]. HR remained unchanged during HUT (*P* > 0.05). SV in the patients (*n* = 8) decreased by about 24% at 1st and 15th min of HUT from baseline (*P* < 0.036), but no significant differences between the groups (*P* > 0.234). CO decreased by 24% at 1st and 15th min compared from baseline (*P* = 0.036) only in patients before surgery, while there were no significant different between the groups. EF significantly decreased by 8% at 15th min compared from 1st min in patients before surgery (*P* = 0.017).

**Table 3:** Heart rate and blood pressure responses before and during 60º head-up tilt

	Group	Baseline	HUT3	HUT6	HUT9	HUT12	HUT15
HR beats/min	Healthy	74 (63–75)	79 (71–83)^†^	79 (71–81)^†^	81 (74–83)^†^	79 (74–84)^†^	79 (73–81)^†^
	Before	66 (59–71)	66 (66–79)	70 (65–74)	68 (66–78)	68 (66–80)	70 (65–78)
	After	75 (61–84)	84 (69–89)**^†^	83 (68–88)**^†^	84 (67–87)**^†^	85 (67–90)**^†^	85 (69–89)**^†^
MAP mmHg	Healthy	89 (86–99)	95 (81–105)	89 (82–102)	88 (85–104)	90 (85–100)	93 (84–104)
	Before	91 (90–103)	93 (90–95)	95 (82–96)	85^†^ (83–98)	91^†^ (84–96)	95 (88–96)
	After	89 (88–90)**	80 (79–84)**^†^	78 (75–82)**^†^	79 (77–82)**^†^	78 (74–85)**^†^	80 (76–83)*^†^
SBP mmHg	Healthy	123 (116–140)	120 (114–143)	118 (113–143)	116 (114–137)^†^	119 (112–134)^†^	124 (114–143)
	Before	138 (133–149)	139 (129–143)	134 (130–144)	126 (125–137)^†^	131 (122–139)^†^	130 (125–141)^†^
	After	121 (116–131)**	113 (106–121)**^†^	108 (106–114)**^†^	110 (102–112)**^†^	105 (99–114)**^†^	107 (102–117)**^†^
DBP mmHg	Healthy	76 (69–79)	82 (65–85)	74 (67–80)	75 (71–84)	76 (71–80)	77 (69–81)
	Before	76 (66–80)	69 (67–77)	70 (64–78)^†^	69 (64–72)^†^	73 (66–74)^†^	73 (66–77)
	After	70 (64–75)	66 (63–70)	63 (61–67)*^†^	64 (57–67)^†^	63 (60–68)	65 (61–68)

Values are medians (1st–3rd quartiles) of 9 patients before (Before) and after CABG (After) and 7 in the healthy subjects (Healthy). Baseline, at rest in supine position before head-up tilt (HUT); HUT3, -6, -9, -12, -15; 3rd, 6th, 9th, 12th, 15th min of HUT

*HR* heart rate, *MAP* mean arterial pressure, *SBP and DBP* systolic and diastolic blood pressures, respectively

* vs. “Healthy”; ** vs. “Before”; ^†^vs. “Baseline” within the same group of subjects

**Table 4:** Echocardiography data before and during 60º head-up tilt

	Group	Baseline	HUT1	HUT15
SV, mL/beat	Healthy	67.9 (54.1–85.8)	66.7 (52.2–87.0)	56.7 (50.1–79.3)
	Before	75.3 (68.5–79.5)	60.2 (40.3–62.8)^†^	54.0 (47.3–58.2)^†^
	After	67.9 (51.8–72.5)	48.1 (33.1–53.9)	50.2 (39.2–52.4)
CO, L/min	Healthy	4.4 (3.2–6.5)	4.3 (3.6–7.1)	4.2 (3.4–6.4)
	Before	4.6 (3.9–5.3)	3.4 (2.7–4.3)^†^	3.6^†^ (3.2–4.0)
	After	4.0 (3.6–4.8)	3.2 (2.7–4.4)	3.6 (3.3–3.9)
TPR, a.u	Healthy	19.8 (13.0–32.5)	19.6 (12.4–30.3)	22.9 (14.7–28.7)
	Before	19.9 (17.7–25.2)	25.5 (21.3–36.5)^†^	24.8 (22.7–28.6)
	After	20.6 (17.9–25.1)	25.1 (20.2–29.2)	22.5 (19.6–23.9)
EF, %	Healthy	67.7 (66.6–72.3)	71.4 (67.2–75.5)	69.0 (66.7–72.8)
	Before	62.2 (57.8–66.3)*	61.2 (57.0–65.8)*	59.2 (44.7–62.4)^$^
	After	60.6 (48.6–61.8)**	54.2 (50.5–62.3)**	50.4 (47.9–55.8)**
LVESV, mL	Healthy	29 (26–33)	23 (22–28)	25 (24–27)^†^
	Before	45 (40–56)*	32 (30–44)^†^	35 (32–45)*^†^
	After	45 (39–59)**	36 (29–40)**^†^	41 (32–48)**^†^
LVEDV, mL	Healthy	97 (82–122)	97 (71–113)	84 (74–105)
	Before	120 (112–133)	93 (77–102)^†^	94 (86–113)^†^
	After	117 (100–127)	84 (65–102)^†^	91 (76–102)^†^

Values are medians (1st–3rd quartiles) of 8 patients before (Before) and after CABG (After) and 7 in the healthy subjects (Healthy). Baseline, at rest in supine position before head-up tilt (HUT); HUT1-15

*SV* stroke volume, *CO* cardiac output, *TPR* total peripheral resistance in arbitrary units (a.u.); *EF* left ventricular ejection fraction, *LVESV* left ventricular end-systolic volume, *LVEDV* left ventricular end-diastolic volume

* vs. “Healthy”; ** vs. “Before”; ^†^vs. “Baseline” within the same group of subjects; ^$^vs. HUT1 at the level of *P* < 0.05

### Responses before and after surgery

A prospective and observational study was done for 9 patients, but that of cardiac echography data was done for 8 patients as mentioned in “Methods” section. MAP before surgery significantly decreased at several time points during HUT from the baseline. After surgery, the baseline MAP was significantly lower by 3 (3–12) mmHg than before surgery (Table 3) and further decreased by 6 (2–13) mmHg during HUT15 from the baseline (*P* = 0.013; Fig. 1). Changes in SBP exhibited a pattern similar to those of MAP. Although HR remained unchanged before surgery, HR significantly increased by 8 (7–11) beats/min at HUT15 from the baseline after surgery (*P* = 0.008; Fig. 1). There were no significant differences in baseline SV, CO, and TPR between before and after surgery. Significant decreases in SV and CO and an increase in TPR during HUT before surgery disappeared after surgery. LVESV and LVEDV decreased by 11 (0–14) and 36 (8–40) at HUT15, respectively, during HUT before surgery and similar decreases were observed after surgery (*P* < 0.02).

### Differences between healthy subjects and patients after surgery

MAP at HUT15 and DBP at HUT6 in the patients after surgery were significantly lower than healthy subjects (Table 3) and EF in the postoperative subjects were lower than healthy subjects because of lower LVESV (Table 4; all *P* < 0.05).

### Blood analysis

[Alb]_s_, [Na^+^]_s_ and Ht were 4.2 (4.0–4.2) g/dL, 141 (138–142) mEq/L and 39 (36–43)% [mean ± SEM], respectively, before surgery, and significantly decreased by 0.9 (0.4–1.1) g/dL, 2.0 (2.0–4.0) mEq/L and 6.4 (0.0–9.7)% after surgery in 9 patients (all *P* < 0.012).

## Discussion

We compared BP response and cardiac function between CAD patients and age- and physical characteristics-matched healthy subjects and assessed effects of CABG and rehabilitation therapy on these responses. The main findings were (1) HUT was associated with a decrease in MAP, while HR remained unchanged in the patients group only, (2) the HUT-related decrease in MAP was enhanced after surgery compared with before surgery, although surgery had effect on HUT-related increase in HR, and (3) surgery had no effect on the HUT-related fall in LVEDV, whereas surgery was followed by decreases in body weight, [Alb]_s_ and [Na^+^]_s_. A deprivation of body fluid would be a prime candidate for the reason.

### Blood pressure in healthy subjects and patients before CABG

In healthy subjects, baroreflexes ensure steady-state and stable arterial blood pressure, even during orthostatic stress [25, 26]. The pressor response to a given increase in sympathetic nerve activity is attenuated with reduction in venous return to the heart by gravitational fluid shift toward the lower extremity [25–27]. However, the increase in HR and enhancement of sympathetic nerve activity through baroreflexes compensate for the attenuation, resulting in restoration of CO and TPR. Furthermore, if the sensitivity of BP to elevated sympathetic nervous activity during HUT increased compared with the supine position, no significant decrease in BP could occur even in the presence of an attenuated HR response to a hypotension.

In the present study, SV remained unchanged during HUT in healthy subjects. We applied HUT with 60˚ inclination using a foot board, not passive tilt without contact of the sole. It was previously demonstrated that active standing was associated with static isometric leg-muscle contraction and high BP, compared with passive tilt, in which blood tends to pool in the lower limb veins after 1 to 7 min [28]. Also, active standing immediately induces a rise in intra-abdominal pressure by about 40 mmHg [29]. In healthy subjects, these mechanisms may mitigate a reduction of venous return to the heart in the standing posture, with a resultant maintenance or increase in SV even during that posture [29].

The baseline BP was similar in both groups, but the HR response to a decrease in BP during HUT was diminished in the patients. Although we did not assess the sensitivity of HR and sympathetic nerve activity response to changes in BP in the present study, a previous study used the phenylephrine technique and found that the baroreflex gain in patients with myocardial infarction and EF of < 40% was lower than patients with EF > 40% [30] and that the gain was reduced by approximately 30% and 40% in CAD patients with and without left ventricular dysfunction, respectively [31]. In our study, attenuation of the increase in cardiac contraction during the fall in BP during HUT was associated with significant decreases in both SV and CO. This should explain the tendency for the larger decrease in BP during HUT in the patient group before surgery, compared with healthy group. Previously, left ventricular diastolic dysfunction was generally observed after myocardial infarction [32] but systolic function was improved after CABG if EF was less than 50% before CABG [33]. However, in the present study, LVESV was higher in the patients before surgery, while LVEDV was similar, suggesting that systolic dysfunction preferred to be greater than diastolic dysfunction. Increases in TPR during HUT in the patients before CABG would be associated with an enhancement of an increase in sympathetic nerve activity to HUT [34, 35] and reduced CO during HUT in the patients.

### Blood pressure in patients before and after surgery

A decrease in BP after surgery further strengthened during HUT relative to that recorded before surgery, because hypovolemia might progress after CABG while cardiac function did not improve enough. Postoperative management involves procedures that prevent body-fluid overload, such as treatment with circulatory agonists and diuretics and limited intravenous fluid infusion [14–19]. Consequently, body weight decreased significantly by about 3 kg, relative to before surgery. Such reduction corresponds to body-fluid loss with probable reduction in venous return to the heart. However, our results showed similar levels of LVEDV before and after surgery (Table 4), suggesting that the reduction in venous return to the heart seems to be limited after surgery.

Previous studies showed higher compliance of lower leg veins after CABG compared with before surgery [36], due to the release of vasoactive substances [37]. The magnitude of change in calf volume during venous occlusion correlated with filling of the deep veins, but the contribution of the excised saphenous vein used for grafting was minor after surgery [38]. The higher compliance could be related with a reduced venous return to the heart even there was no significant decrease in SV during HUT after CABG.

A previous study that analyzed HR variability before and after CABG showed a decrease in high frequency power to one-third of the preoperative level at 1 week after surgery [34], suggesting that sympathetic nerve activity was dominant relative to parasympathetic nerve activity, compared to before surgery. Since the postoperative measurements were conducted in our study at a time period similar to that in the above study, we presume that sympathetic activity was enhanced also in our patients after surgery. Plasma norepinephrine concentrations at rest, which is used as an index of sympathetic nerve activity, were significantly higher at 1 week after CABG but diminished at 3 weeks after surgery, while baroreflex sensitivity declined further [35]. These results suggest impairment of BP control through baroreflexes after CABG and such impairment could be enhanced at least until 3 weeks after surgery, despite the gradual increase in sympathetic nerve activity during hospital stay.

The reason why the increase in TPR during HUT disappeared after CABG was initiated by the higher compliance of the lower leg veins compared with before surgery [36]. Sympathetic nerve activity itself was likely to be enhanced after surgery as mentioned above [35]; furthermore, the higher compliance could reduce venous return to the heart, resulting in enhancing sympathetic nerve activity via baroreceptors unloading [8]. However, baroreflex sensitivity would be attenuated after surgery [9, 10]. The effects exceeded the vasoconstrictor activity induced by sympathetic nervous system. Releases of vasodilatory substances after surgery in the whole body [37] might be also associated with the disappearance.

Six patients had old myocardial infarction while three patients had unstable angina pectoris. The difference in pathological conditions between both diseases could influence on the present results. Averaged EF in patients with myocardial infarction was 54% and that angina pectoris was 67%, 13% lower in patients with myocardial infarction than that with angina pectoris [39]. However, EF were similar between the 6 myocardial infarction and the 3 unstable angina pectoris in the present study. A previous report suggested that baroreflex sensitivity during a sequence method was attenuated in patients with both diseases similarly compared with that in the age-matched healthy persons [31]. These results suggest that pathological backgrounds, e.g., atherosclerosis, were similar between these diseases. Thus, BP regulation during HUT would be equivalent between the both diseases if EF would be preserved.

As shown in Table 2, all patients in the present study took at least one antihypertensive before surgery such as β-blocker (*n* = 2), αβ-blocker (*n* = 3), calcium antagonist (n = 6), angiotensin II receptor blocker (*n* = 6) and nitrates (*n* = 2). After surgery, all patients also took at least one antihypertensive; β-blocker (*n* = 6), αβ-blocker (*n* = 6), calcium antagonist (*n* = 7), angiotensin II receptor blocker (*n* = 4), and catecholamines (noradrenaline and dobutamine; *n* = 7), and n = 9 for nitrates, diuretics, and dopamine. Patterns of the prescription before and after surgery varied widely but basically several types of medications were added after surgery. Therefore, we could not deny the possibilities that these antihypertensive itself or changes in patterns of each prescription influenced on the BP response through modified BRS. Moreover, additions of diuretics would be related to the reduced body weight after surgery.

### Advantages of rehabilitation

Hypovolemia was developed just 2–3 days after bed rest, resulting in orthostatic hypotension in healthy subjects [40]. Thus, early mobilization is important to prevent this complication. In our study, SBP decreased by more than 20 mmHg during HUT in 1 out of 9 patients, but fainting or syncope did not occur in the present study. Exercise program for 6 weeks, which was conducted 3 months after CABG, significantly improved the reductions in SV and CO during HUT compared with that before the intervention [21]. Considered together, these results highlight the importance of exercise in attenuating any decrease in body-fluid volume. Four-week exercise for CAD patients was associated with 30% improvement in baroreflex sensitivity [41]. Exercise significantly improved cardiovascular responses after CABG [42]. Therefore, improvement in these functions are expected after CABG. Our rehabilitation therapy possibly improved or maintained BP control, as reflected by the absence of any fainting or syncope after CABG in the standing position.

LVEDV after surgery was maintained at a level similar to that recorded before surgery, despite the expected postoperative body-fluid loss (Table 4). Previous studies showed reduced peripheral edema of donor limb after CABG in patients with increased daily activity [43]. Although we did not assess a lower limb volume due to edema, a reduction in peripheral edema based on rehabilitation therapy and/or physical activity possibly contributed to the maintenance of LVEDV after surgery.

### Limitations

This is a preliminary prospective observational study. Even we excluded biases of aging and body compositions, there were some variations of prescribing medications and all patients did not stay for the same time period. All patients had an elective operation (not an emergency operation) and an isolated CABG surgery from a single facility. Sample size was small and non-parametric tests were conducted because some of data were not normally distributed. Furthermore, healthy subjects also needed to be tested twice with a similar interval to the patients, because the patients were possibly nervous during the first test but it was relieved during the second time. The present results could be reproduced in limited populations.

Body-fluid loss after CABG as expected could affect cardiovascular dysfunction in the patients. However, rehabilitation therapy in the present study might not retain the loss completely, even we started it early. To compensate this dysfunction in CABG patients, the other strategy is needed to expand body fluid before CABG, which can be achieved by endurance training with protein supplementation [44].

## Conclusion

We have demonstrated a decrease in BP during HUT due to a decrease in CO because of a decrease in SV without an increase in HR in CAD patients, whereas BP remained unchanged with the increased HR in the healthy group. The decrease in BP was augmented after CABG despite the improvement of HR response. Even the lack of syncope during the hospital stay was possibly related to the early post-CABG rehabilitation therapy, a preoperative rehabilitation would be needed to compensate postoperative cardiovascular dysfunction in the patients.

## Abbreviations

[Alb]_s_: Serum concentration of albumin
BP: Blood pressure
CABG: Coronary artery bypass grafting
CAD: Coronary artery disease
CO: Cardiac output
EF: Ejection fraction
HR: Heart rate
HUT: Head-up tilt
LVEDV and LVESV: Left ventricle end-diastolic and end-systolic volumes
MAP: Mean arterial pressure
[Na^+^]_s_: Serum concentration of sodium
POD: Postoperative day
SBP and DBP: Systolic and diastolic blood pressures
SV: Stroke volume
TPR: Total peripheral vascular resistance

## Availability of data and materials

The datasets used and/or analyzed during the current study are available from the corresponding author on reasonable request.
